# Genomic and phenotypic characterization of methicillin-resistant *Staphylococcus aureus* ST965: an emerging hospital-adapted clone with enhanced invasiveness

**DOI:** 10.1128/msystems.00798-25

**Published:** 2025-07-31

**Authors:** Jianbo Lv, Zhixuan Chen, Can yang, Yu Huang, Xinru Yuan, Li Shen, Cailin Wan, Peiyao Zhou, Haojin Gao, Weihua Han, Junhong Shi, Bingjie Wang, Fangyou Yu

**Affiliations:** 1 School of Public Health, Nanchang University 47861, Nanchang, Jiangxi, China; 2 Department of Clinical Laboratory, Shanghai Pulmonary Hospital, School of Medicine, Tongji University 12476, Shanghai, China; Zhejiang University School of Medicine, Hangzhou, Zhejiang, China

**Keywords:** MRSA, ST965, epidemiology, phylogenetic analysis, invasiveness

## Abstract

*Staphylococcus aureus* sequence type 965 (ST965) represents a significant member of clonal complex 5. However, there are still significant gaps in research regarding the resistance, virulence, and distribution characteristics of methicillin-resistant *S. aureus* (MRSA) ST965. Here, we conducted a comprehensive analysis of 27 ST965-MRSA genomes from three different countries, including 20 genomes sequenced in this study and 7 genomes retrieved from public databases. Our results indicate that ST965-MRSA mainly originates from China (92.6%, 25 out of 27) and was first reported in 2013. SCC*mec* IV typing (75%, 21 out of 27) and *spa* t062 typing (75%, 21 out of 27) constituted its predominant clonal characteristics. Among ST965-MRSA clinical isolates, bloodstream infection samples accounted for 35%. A novel multidrug-resistant plasmid (pYF965) was identified in ST965-MRSA, which contains *erm* genes integrated via Tn551 into pTZ2162 and simultaneously contains *aac(6′)-Ie* and *blaI-blaR-blaZ*. Through the acute pathogenicity model, ST965-MRSA exhibited higher hemolytic activity and mortality rates compared to N315 (ST5), yet their overall virulence remained significantly lower than the highly virulent strain USA300-LAC (ST8) (*P <* 0.0001). Notably, in terms of persistent infection, the bacterial colonization levels of ST965-MRSA in the heart, lungs, and spleen *in vivo* were significantly higher than those of N315 (ST5) and USA300-LAC (ST8) (*P <* 0.05). Particularly, its immune evasion ability and intracellular survival ability were superior to those of ST5 and ST8 (*P <* 0.05). This study elucidated the biological characteristics of ST965-MRSA for the first time, expanding our understanding of the molecular epidemiology of the ST965 clone.

**IMPORTANCE:**

As an emerging clonal lineage, ST965 has been predominantly discovered in Asian regions. However, research regarding the ST965-MRSA remains highly limited at present. Therefore, we conducted a detailed analysis of the genomic characteristics of ST965-MRSA and, combined with phenotypic experiments, further revealed their potential pathogenic risks. This study initially uncovers the potential global transmission and genetic and phenotypic features of ST965-MRSA and offers valuable insights for controlling and preventing persistent hospital infections.

## INTRODUCTION

*Staphylococcus aureus* represents a significant human pathogenic bacterium that can induce infections spanning from mild cutaneous and soft tissue manifestations to life-threatening invasive systemic conditions (1, 2). Following the initial appearance of methicillin-resistant *S. aureus* (MRSA) in 1961 (3), the global transmission patterns and molecular evolutionary features of this pathogen have developed into key priorities within public health surveillance (4, 5). MRSA strains exhibit intrinsic resistance to β-lactam antibiotics and are frequently associated with multidrug-resistant phenotypes, posing significant challenges to clinical treatment strategies (6–8). According to World Health Organization data, patients with MRSA infections exhibit a mortality rate 64% greater than those with other infectious etiologies (9–11).

To enhance our understanding of *S. aureus* epidemiology, investigators have established multiple molecular typing methods. Among these, multilocus sequence typing (MLST) has been widely applied (12). MLST analyzes internal fragments of seven housekeeping genes and determines the ST based on the unique combination of alleles (13). Using MLST, investigators have characterized numerous prevalent MRSA clonal lineages, including ST5, ST239, and ST59 (14–16), which demonstrate geographic distribution specificities and variations in clinical phenotypes (17). As technologies advance and clinical monitoring intensifies, researchers continue to discover and report novel sequence types (18).

*Staphylococcus aureus* sequence type 965 (ST965) represents a comparatively rare sequence type initially identified and documented in the Asian region in 2013 (19). ST965, along with ST5 and ST764, forms principal branches within clonal complex 5 (CC5) (20). Notably, ST965 has been identified in both MRSA and methicillin-susceptible *S. aureus* (21). ST965-MRSA harbors the SCC*mec* type IV element and has been isolated from cases of both skin and soft tissue infections, as well as bloodstream infections (22). Despite these initial characterizations, critical attributes, including genomic characteristics, virulence factors, and antimicrobial resistance mechanisms, remain to be clarified.

Given these considerations, this investigation collected 20 ST965-MRSA isolates over 7 years from seven provinces in China, performed phylogenetic analysis, and comparatively analyzed mobile genetic elements, drug resistance, and virulence factors between ST965 and globally representative MRSA (ST5, ST8, and ST1) to examine the genetic features of ST965-MRSA. Through the combination of phenotypic experiments, we aim to elucidate the genetic characteristics and phenotypic features of ST965-MRSA, thereby contributing to the development of infection prevention and control measures.

## MATERIALS AND METHODS

### Bacterial isolates and growth conditions

A total of 565 non-duplicate clinical MRSA isolates were collected between 2014 and 2020 from seven provinces and municipalities across China: Guangdong (*n* = 112), Zhejiang (*n* = 108), Sichuan (*n* = 95), Shanghai (*n* = 84), Hubei (*n* = 70), Jiangxi (*n* = 58), and Inner Mongolia Autonomous Region (*n* = 38) (23). All isolates were confirmed with the cefoxitin disk diffusion test. MLST analysis revealed that 20 isolates (3.5%, 20 out of 565) belonged to ST965. These ST965 isolates were selected for further detailed investigation. In this study, *S. aureus* isolates were cultured in tryptic soy broth (TSB) (Oxoid, UK) with shaking at 37°C and 220 rpm.

### Whole-genome sequencing and analysis

Whole-genome sequencing (WGS) of 20 ST965-MRSA isolates was performed on an Illumina NovaSeq platform. Sequence reads were quality-filtered using fastp (v.0.20.1) (24) and *de novo* assembled using Unicycler (v.0.5.0) (25). MLST (v.2.19.0, https://github.com/tseemann/mlst) was employed for multilocus sequence typing, while SCC*mec*Finder was used to identify SCC*mec* types (26). To investigate phylogenetic relationships, genome sequences of 12 ST965 strains were retrieved from the NCBI RefSeq database (as of April 2025). Core genome single-nucleotide polymorphism (SNP) analysis was performed using Snippy (v.4.6.0) and Gubbins (v.2.3.4) with M3912 (GenBank accession number GCF_900251875) as the earliest discovered reference (27). Phylogenetic trees were visualized using iTOL (28). AntiSMASH (v.7.0.2) was employed to predict secondary metabolite biosynthetic gene clusters (BGCs) (29). The presence of genomic islands and prophages was investigated using BLASTN searches, confirming hits that exhibited more than 80% sequence similarity and coverage, followed by manual verification to ensure accuracy. Comparisons of mobile genetic elements were visualized using the gggenomes package (v.0.9.5.9000, https://github.com/thackl/gggenomes) in R (30). Illumina sequencing data of all ST965 isolates have been submitted to the NCBI database (accession number PRJNA1245302).

### Antimicrobial susceptibility testing

This study assessed the sensitivity of ST965-MRSA isolates to 13 antimicrobial drugs. Antimicrobial susceptibility to cefoxitin, erythromycin, tetracycline, and ciprofloxacin was tested using the disk diffusion method on Mueller-Hinton agar plates (Oxoid). Minimum inhibitory concentrations (MICs) of gentamicin, daptomycin, mupirocin, rifampin, teicoplanin, linezolid, fusidic acid, vancomycin, and dalbavancin were measured using the broth microdilution method. All antimicrobial susceptibility testing and result interpretation were conducted according to the breakpoint criteria in the 2025 guidelines of the Clinical and Laboratory Standards Institute (CLSI). The MIC of fusidic acid was interpreted according to the European Committee on Antimicrobial Susceptibility Testing clinical breakpoints (31). American Type Culture Collection (ATCC) 25923 and ATCC 29213 served as quality control strains for the disk diffusion method and broth microdilution method, respectively.

### Cell invasion assay

Bacterial cultures were diluted in serum-free Dulbecco’s Modified Eagle Medium (DMEM) to an OD_600_ = 0.5 (equivalent to approximately 1 × 10⁸ CFU/mL). A549 cells were seeded in 12-well plates at a density of 5 × 10⁵ cells/well and cultured to 80%–90% confluence. Following two phosphate-buffered saline (PBS) washes, cells were infected at a multiplicity of infection of 10:1 and co-cultured at 37°C in 5% CO_2_ atmosphere for 1 hour. After aspirating the supernatant, cells were treated with DMEM supplemented with 10 µg/mL lysostaphin and 100 µg/mL gentamicin for 1 hour to eliminate extracellular bacteria. Following three PBS washes, cells were further incubated in DMEM supplemented with 10% FBS. At 12 hours post-infection, cells were washed three times with PBS, and 1 mL of 0.05% Triton X-100 was added to lyse cells at 37°C for 10 minutes. The lysate was serially diluted and plated onto tryptic soy agar (TSA) plates, followed by incubation at 37°C for 18 hours before enumeration of CFU.

### Human whole blood killing assay

Overnight cultures were collected. Bacteria were pelleted by centrifugation at 4,000 rpm for 10 minutes, washed three times with sterile PBS (pH 7.2), and adjusted to 1 × 10⁶ CFU/mL. Bacterial suspensions were mixed with healthy volunteer whole blood at a 1:9 ratio and incubated at 37°C with shaking at 220 rpm. Samples were taken at 0 hours (inoculation point) and 3 hours and subjected to 10-fold serial dilutions, and appropriate dilutions were plated on TSA plates, with colonies counted after 18 hours of incubation at 37°C.

### Hemolytic activity assay

The bacterial cultures were collected after centrifugation at 4,000 rpm for 10 minutes. The bacterial culture supernatant was mixed with 900 µL of PBS containing 3% defibrinated rabbit erythrocytes and incubated at 37°C for 1 hour. Triton X-100 served as the positive control for 100% hemolysis, while PBS served as the negative control. After centrifugation, supernatants were collected and quantified by recording the OD_600_ of each well.

### Mouse skin abscess model

Female BALB/c nude mice (6 weeks old, SPF grade) were randomly allocated into six groups (*n* = 5 per group) and maintained under standard environmental conditions for 1 week of acclimatization. MRSA ST965 and USA300-LAC strains were cultured overnight in TSB at 37°C with shaking, collected by centrifugation at 4,000 rpm for 10 minutes, washed three times with PBS, and adjusted to 1 × 10⁸ CFU/mL. Each mouse received a subcutaneous injection of 100 µL bacterial suspension (1 × 10⁷ CFU), while control groups were injected with an equal volume of PBS. Skin lesions were observed and recorded daily, with abscess length (*L*) and width (*W*) measured using digital calipers to calculate the area: *A* = *L* × *W* (mm²).

### Mouse bloodstream infection model

The isolates were cultured in TSB at 37°C for 16 hours, washed three times with sterile PBS, and finally resuspended in PBS with bacterial concentration adjusted to 1 × 10^8^ CFU/mL. Mice were administered 100 µL of the bacterial suspension via tail vein injection. The survival status of mice was monitored continuously over 48 hours, with mortality time points documented and survival curves constructed. Hearts, livers, spleens, lungs, and kidneys were harvested from deceased mice and surface-rinsed with sterile PBS. Multi-sample Tissue Grinder-24L (Shanghai Jingxin) was used to prepare tissue homogenates. Tissue homogenates were serially diluted and plated on TSA plates, followed by incubation at 37°C for 18 hours before colony counting.

### Statistical analysis

All data were statistically analyzed and graphically presented using GraphPad Prism 9.0 software (GraphPad Software Inc., San Diego, CA, USA). Results derived from samples between two groups were treated with unpaired two-tailed Student’s *t*-test and *χ*^2^ test. *P* < 0.05 was statistically considered to be significant (*P < 0.05, **P < 0.01, ***P < 0.001, and ****P < 0.0001).

## RESULTS

### Demographic characteristics of ST965-MRSA isolates

**Fig 1:**
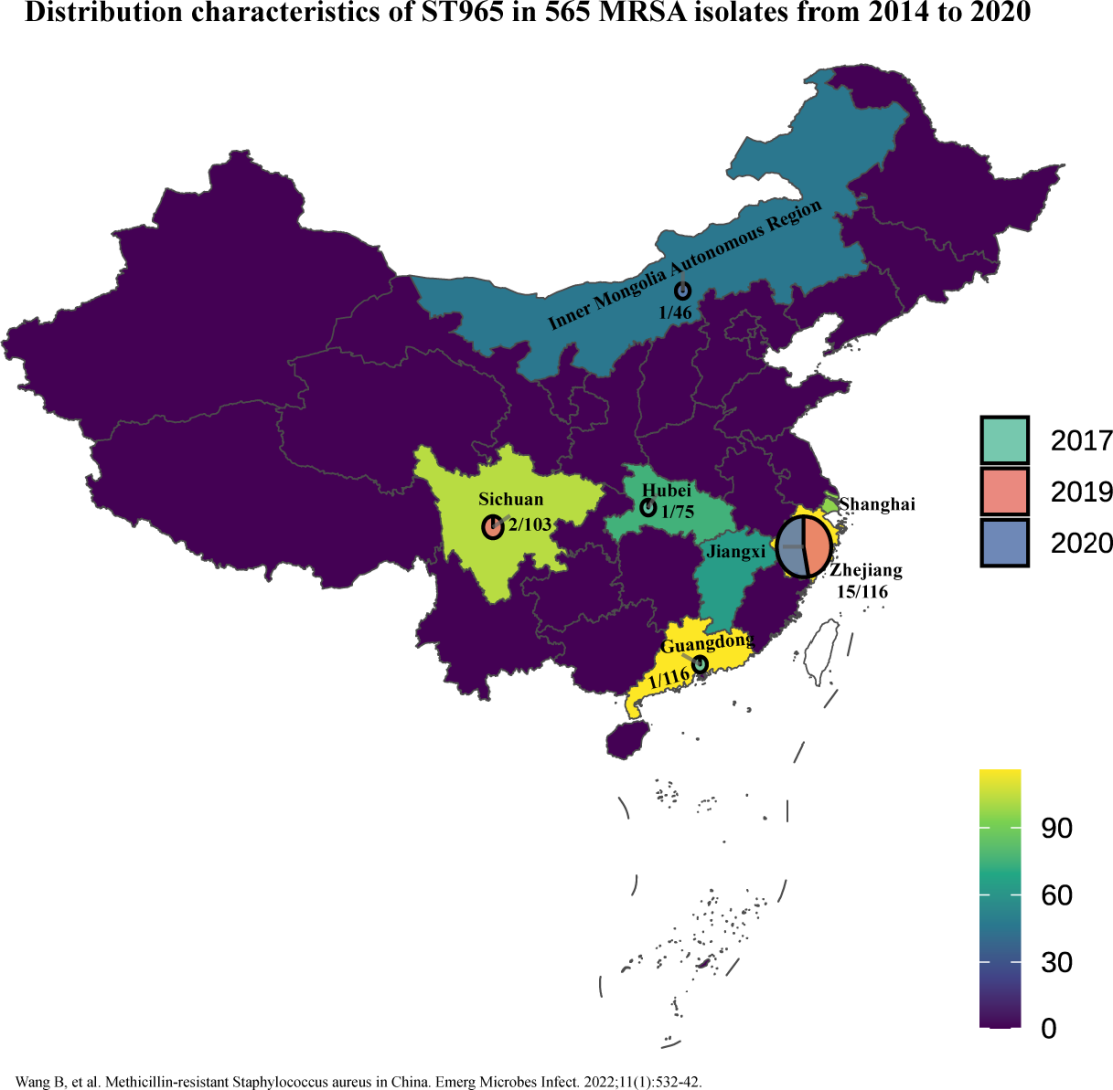
Geographic distribution of 20 ST965-MRSA among 565 MRSA clinical isolates over 7 years from seven provinces in China.

As shown in Fig. 1, the geographic distribution of these ST965-MRSA isolates was predominantly in Zhejiang Province (75%, 15 out of 20), with the remainder distributed in Sichuan Province (10%, 2 out of 20), Inner Mongolia Autonomous Region (5%, 1 out of 20), Hubei Province (5%, 1 out of 20), and Guangdong Province (5%, 1 out of 20). Regarding sample sources, the clinical specimens were sourced from blood (35%, 7 out of 20), pus (35%, 7 out of 20), and sputum (30%, 6 out of 20). In terms of patient demographics, males constituted the majority of patients (70%, 14 out of 20). When examining the temporal distribution, during the 7-year period covered by the study, the isolation rate of MRSA ST965 was mainly in 2019 at 45%.

### Resistance gene analysis and antibiotic susceptibility testing of ST965-MRSA isolates

**Fig 2:**
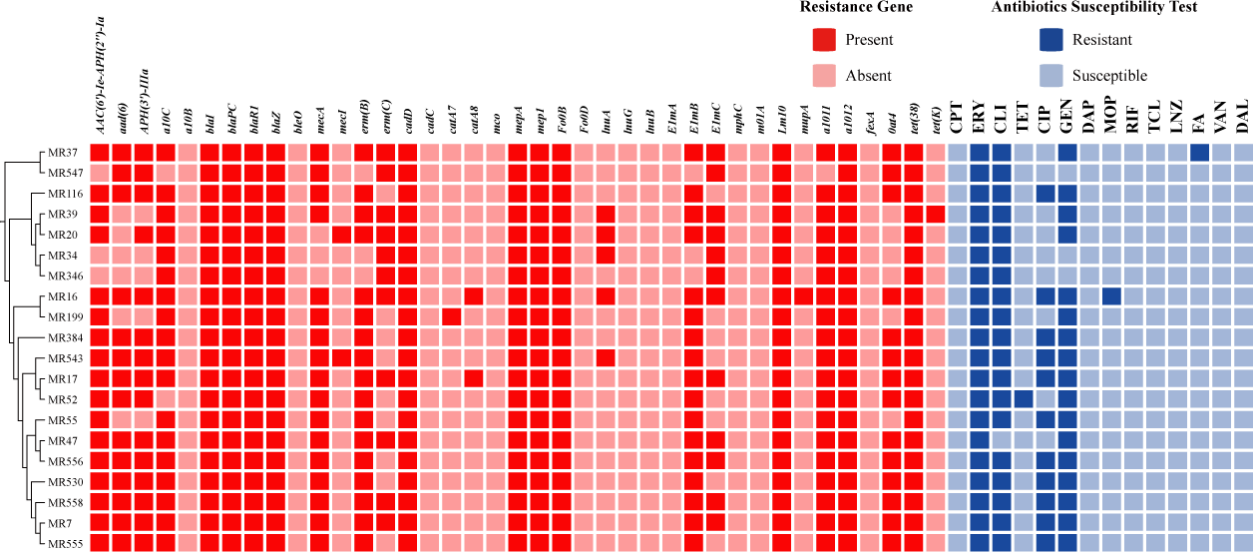
Antibiotic resistance genes (left) and antibiotic susceptibility profiles (right) of 20 ST965-MRSA isolates that were collected in this study.

Results of antimicrobial susceptibility testing (Fig. 2, right) demonstrated that ST965-MRSA isolates exhibit multidrug resistance profiles. The highest resistance rate was observed for erythromycin (100%, 20 out of 20), followed by clindamycin (90%, 18 out of 20), gentamicin (85%, 17 out of 20), and ciprofloxacin (55%, 11 out of 20). All isolates remained susceptible to ceftaroline, daptomycin, rifampin, teicoplanin, linezolid, dalbavancin, and vancomycin, with MIC values below the resistance breakpoints specified by CLSI. To elucidate the genetic basis of these resistance patterns, resistance gene detection (Fig. 2, left) further revealed the molecular basis of multidrug resistance in these isolates. The β-lactam resistance regulatory gene *blaI-blaR-blaZ* was detected in all isolates. Erythromycin and clindamycin resistance was significantly associated with the presence of *erm(B)* and *erm(C)*. Carriage of aminoglycoside resistance genes *aac(6′)-Ie-aph(2″)-Ia* and *aph(3')-IIIa* demonstrated strong concordance with gentamicin resistance phenotypes. Moreover, we detected the multidrug efflux pump gene *mepA* and its regulatory gene, *mepR*, along with *cadD* and *cadC* operons related to heavy metal ion efflux, which may collectively confer broader antibiotic tolerance to these isolates.

### Virulence genes analysis of ST965-MRSA

**Fig 3:**
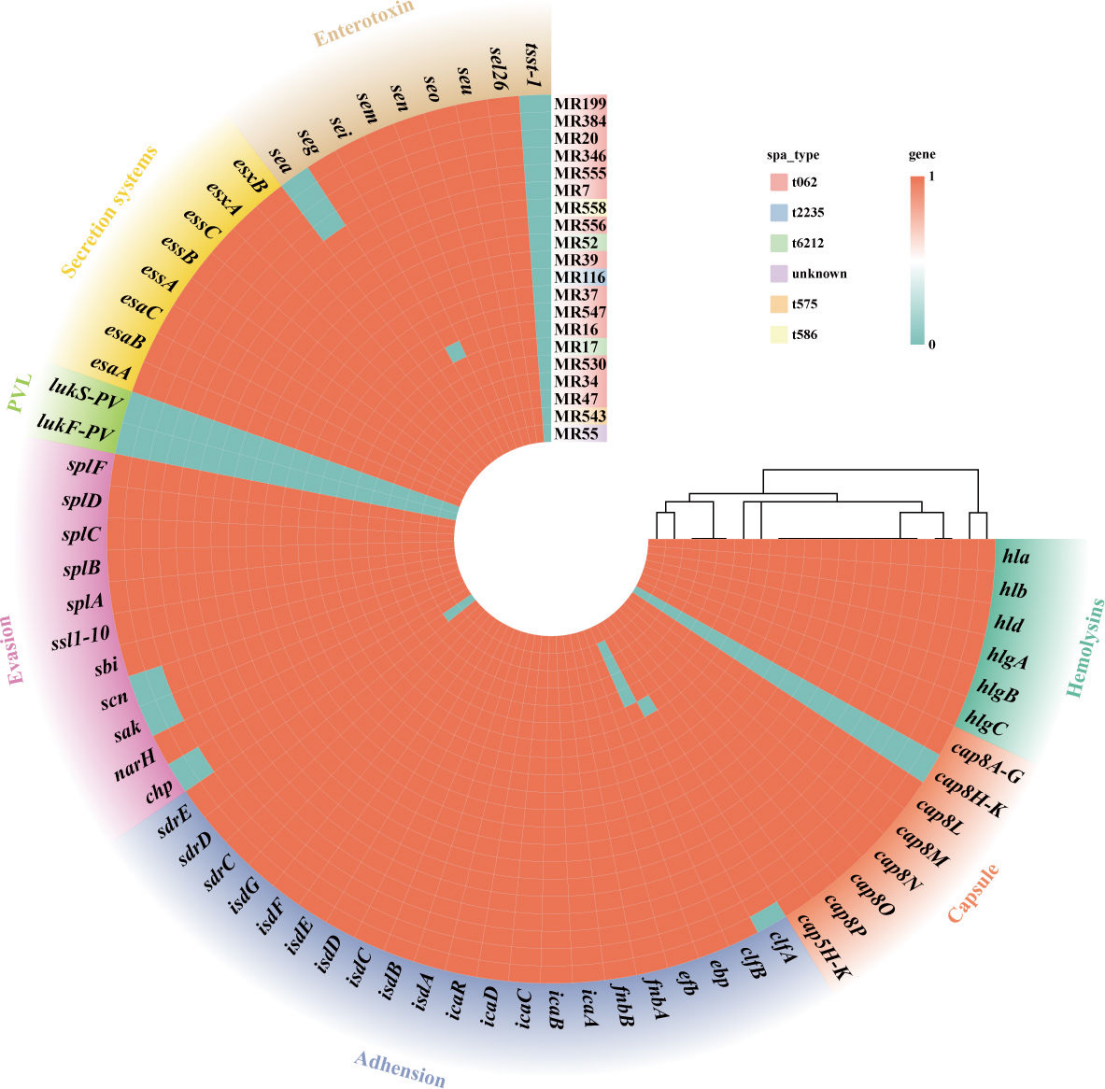
Virulence gene profiles of 20 ST965-MRSA clinical isolates. Colored regions in clockwise order represent genes associated with hemolysins, capsule, adhesion, PVL, secretion systems, exoenzymes, and enterotoxins.

As shown in Fig. 3, all ST965 isolates harbored hemolysin-related genes *hla*, *hlb*, *hlg*, and *hld*. However, all isolates lacked both the *lukS/F-PV* encoding Panton-Valentine leukocidin and the toxic shock syndrome toxin, *tsst-1*. In contrast to these toxin deficiencies, the *cap8* series genes and *isd* gene clusters were present in most isolates, indicating that these isolates may have strong antiphagocytic capabilities. Regarding secretion and immune evasion systems, all ST965 isolates possessed complete secretion systems (*esa*, *ess*, and *esx*), while partial enterotoxin genes (*sea*, *seg*, *sei*, *sem*, *sen*, *seo*, and *seu*) were highly conserved in ST965, and genes involved in immune evasion (*sak*, *scn*, *sbi*, *ssl*, and *spl*) were present in most isolates, collectively suggesting that the ST965-MRSA profile may be oriented toward persistence.

### Phylogenetic and comparative genomic analysis of ST965

**Fig 4:**
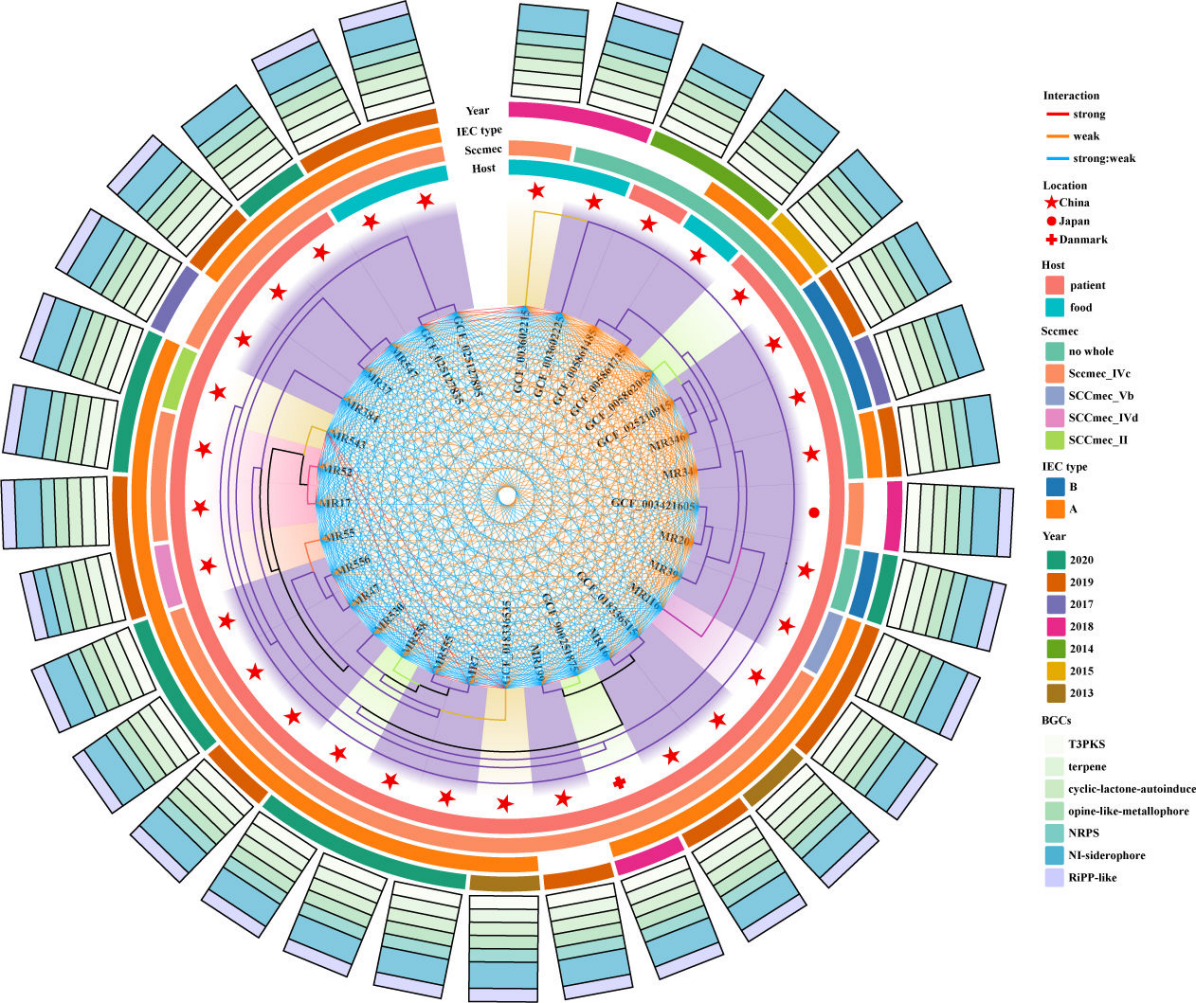
Phylogenetic analysis of 32 ST965 isolates. Different colored branches in the tree diagram represent different *spa* types of the strains. From the inner to the outer circle, isolation location, host source, SCC*mec* type, IEC type, isolation date, and distribution of biosynthetic gene clusters (BGCs) are displayed sequentially.

To explore the evolutionary history of ST965 isolates, we constructed a phylogenetic tree based on core SNPs. The analysis included 20 ST965-MRSA isolates collected in this study and 12 ST965 genomes (including 7 MRSA and 5 MSSA) from the National Center for Biotechnology Information (NCBI) RefSeq database (data current as of April 2025). Phylogenetic analysis showed (Fig. 4) that the earliest identified ST965 isolate could be traced back to China in 2013, with this region accounting for the vast majority of isolate origins (93.75%, 30 out of 32). Despite limited international spread, one ST965 isolate was identified in Denmark and another in Japan, indicating that ST965 has emerged internationally but remains predominantly prevalent in China. When examining the temporal distribution, early samples (2013–2015) were relatively few, while samples from 2019 to 2020 were more concentrated. These samples were predominantly sourced from patients and food, highlighting their potential epidemiological significance. To further characterize these isolates, molecular typing results demonstrated that SCC*mec* type IV was the predominant type (75%, 15 out of 20), with additional detection of one SCC*mec* type V isolate (5%, 1 out of 20) and one type II isolate (5%, 1 out of 20), while three isolates had incomplete SCC*mec* structures due to insertion sequence element insertions. Similarly, the *spa* type was predominantly t062 (70%, 14 out of 20), followed by the novel *spa* type t6212 (10%, 2 out of 20), with one isolate each of t586 and t575 (5%, 1 out of 20). Analysis of *agr* type revealed that all ST965 isolates belonged to type II. For immune evasion characteristics, immune evasion cluster (IEC) typing was predominantly type A (71.8%, 23 out of 32), with a minority being type B (9.4%, 3 out of 32), and six isolates lacking IEC due to gene deletions. Notably, all ST965 isolates harbored the arginine catabolic mobile element type II element, which contains the *arc* gene cluster that enhances bacterial survival in environments with pH fluctuations and high urea concentrations, potentially facilitating ST965 colonization in hospital environments and on human skin surfaces. Further exploration of microbial genomes has revealed numerous BGCs that encode important compounds such as antibiotics and anticancer drugs. Analysis revealed that all ST965 isolates contained T3PKS (type III polyketide synthases), terpene, cyclic-lactone-autoinducer, opine-like-metallophore, non-ribosomal peptide synthetase, and non-ribosomal iron-siderophore gene clusters. The genetic diversity among strains is mainly reflected in the other unspecified ribosomally synthesized and post-translationally modified peptide product (RIPP-like) gene clusters. Correlating with resistant phenotypes, we observed an association between RIPP-like BGCs and gentamicin resistance, which requires future functional studies to confirm causality.

### Genetic associations and potential transmission events among ST965-MRSA isolates

In order to investigate the spread dynamics of ST965-MRSA isolates in hospitals, this research utilized SNP quantitative analysis in conjunction with epidemiological survey approaches to detect potential hospital-acquired transmission events. Figure S1 demonstrates that SNP variations among isolates were broadly distributed, ranging from 0 to 298 SNPs, suggesting considerable genetic diversity exists within this bacterial community. It is noteworthy that merely 1 SNP difference was identified between isolates MR52 and MR17, substantially below the established transmission correlation threshold (SNP <20) (32), revealing high genetic homogeneity between these two isolates and providing strong evidence of a direct nosocomial transmission chain. Furthermore, both isolates exhibited SNP differences exceeding 75 when compared with other ST965 isolates, providing additional evidence of their distinctive genetic linkage.

### Mobile genetic elements in ST965-MRSA and comparisons with other clones

**Fig 5:**
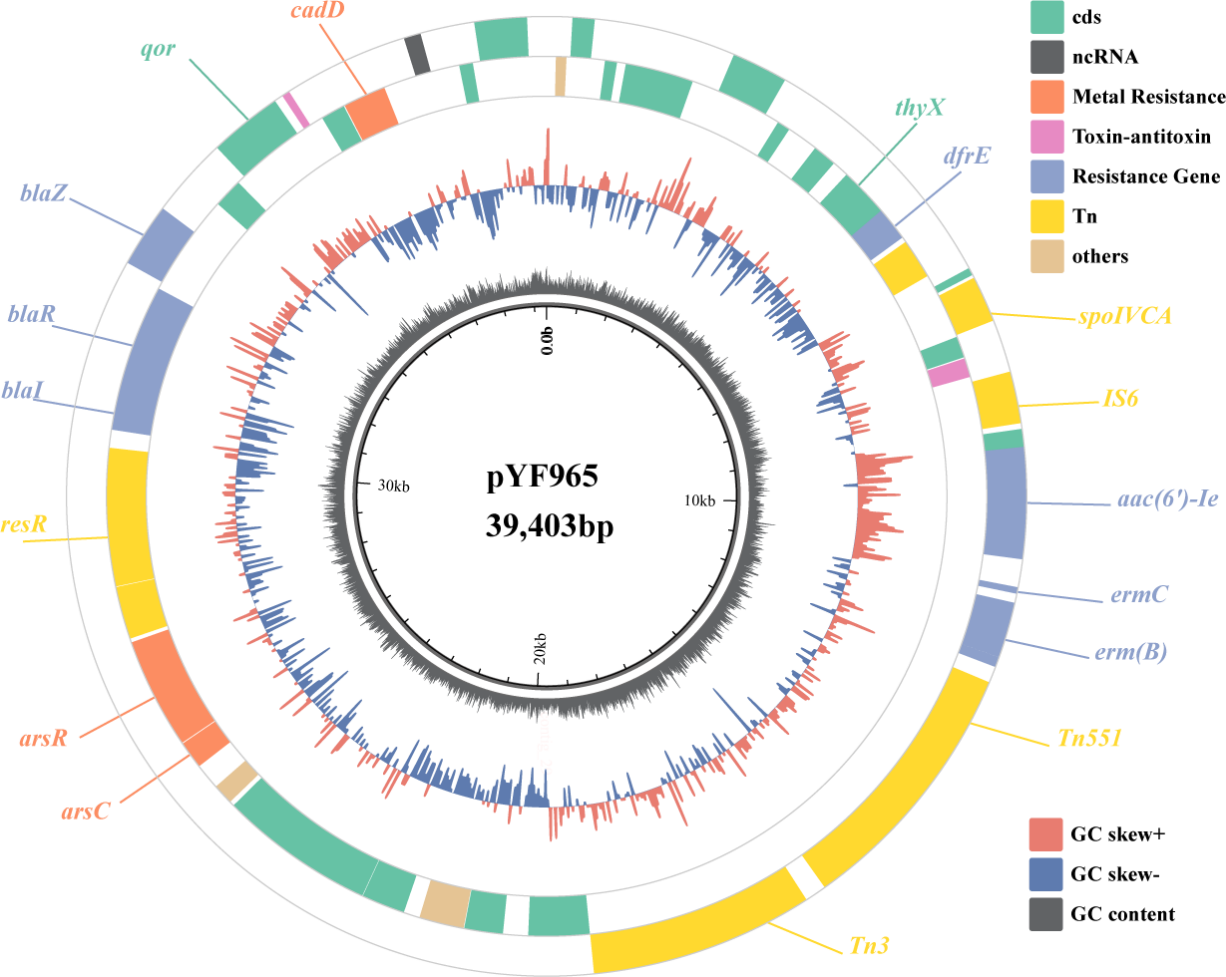
Schematic representation of plasmid pYF965. From inner to outer circles, the rings represent guanine-cytosine (GC) content, GC skew, and coding sequence (CDS). The circularized pYF965 plasmid includes the annotations of the coding sequences (green), resistance genes (blue), metal tolerance (orange), and transposons (yellow).

To investigate the biological background of ST965-MRSA, we identified a plasmid of ~39,000 bp from ST965-MRSA through WGS and named it pYF965. As shown in Fig. 5, it encodes a total of 50 coding sequences, including *aac(6′)-Ie* mediating aminoglycoside resistance, *erm(B)* and *erm(C)* encoding erythromycin resistance, *cadD-cadX* operon conferring cadmium tolerance, beta-lactamase-related genes *blaI-blaR-blaZ*, *arsB-arsC* gene cluster regulating arsenic efflux, and genes encoding lactococcin-type bacteriocins. This plasmid shares extensive identity (99%) with pTZ2162 but additionally contains *erm* genes inserted via the Tn551 transposon, which is a key distinguishing feature in the formation of pYF965. This plasmid confers multiple drug resistance to ST965-MRSA and significantly enhances its adaptability and competitive advantage in hospital environments.

**Fig 6:**
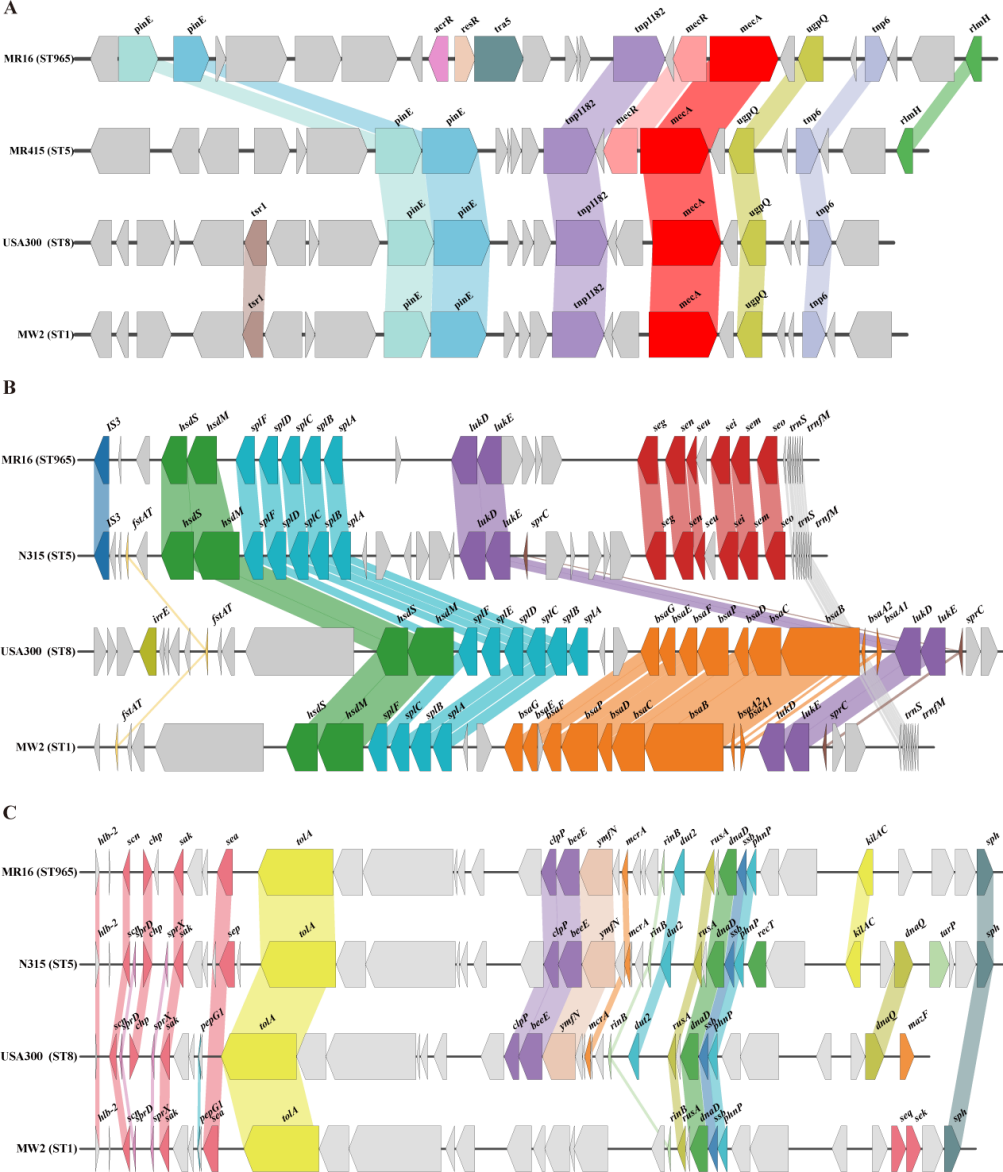
Comparison of pathogenicity islands and prophages among ST965 and ST5, ST8, and ST1 strains. Comparison of the genomic structure of (**A**) SCC*mec*, (**B**) vSaβ, and (**C**) ΦSa3 between ST965, ST8, ST1, and ST5 strains. Arrowed boxes represent genes that are colored according to functional classification.

Analysis revealed that the SCC*mec* type IV carried by ST965-MRSA has the most streamlined structure among all SCC*mec* types. The relatively small SCC*mec* IV (21–24 kb) not only reduces the metabolic burden on strain growth but also significantly improves its horizontal transfer efficiency between MRSA, thereby enhancing transmission potential. Specifically, SCC*mec* IV contains a type B *mec* complex and *ccrA2* and *ccrB2* genes, which encode recombinases responsible for SCC*mec* integration and excision, thereby affecting its mobility and stability between strains. Beyond SCC*mec* elements, all evolutionary branches contain the vSaβ genomic island, which consists of a restriction/modification system (*hsdM*/*hsdS*), a set of serine protease-like genes (*spl*), a group of bacterial surface attachment protein genes (*bsa*), leukotoxin genes (*lukD* and *lukE*), and an enterotoxin gene cluster (*egc*). Comparative genomic analysis revealed that ST965-MRSA lacks the *bsa* gene cluster when compared to ST1 and ST8 sequence types; however, the *egc* gene cluster is present in ST965-MRSA but absent in ST1 and ST8 (Fig. 6B). In addition to these genomic islands, the ΦSa3 prophage containing the IEC was found in 90% (18 out of 20) of ST965-MRSA, which is also known as β-hemolysin converting phage (βC-Φs) because it inserts into the *hlb* gene encoding β-hemolysin during integration. IEC typically contains several key genes, including *chp* (chemotaxis inhibitory protein), *sak* (staphylokinase), *scn* (staphylococcal complement inhibitor), and *sea* (enterotoxin A) in some strains. Functionally, the proteins encoded by these genes can interfere with the host innate immune defense mechanisms. This represents an evolutionary trade-off where, although phage integration leads to the loss of *hlb* gene function, the acquisition of IEC-mediated immune evasion capabilities provides bacteria with a greater selective advantage, especially in the human body environment.

### The virulence of ST965-MRSA is lower than that of USA300-LAC

**Fig 7:**
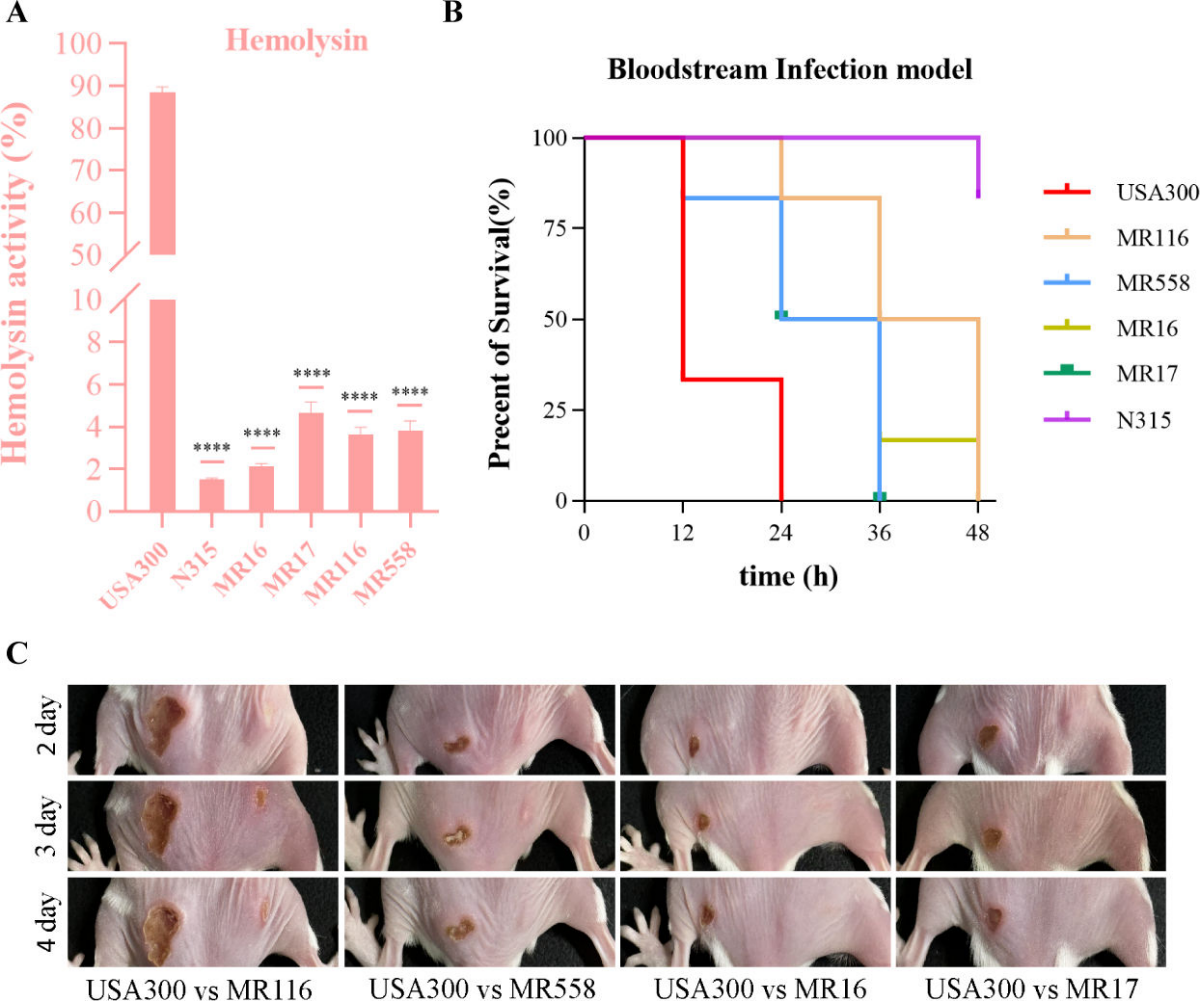
Virulence phenotype of ST965-MRSA from our collection and ST8 (USA300). (**A**) Comparison of hemolytic activity between ST965-MRSA isolates and N315 (ST5) and USA300-LAC (ST8). Triton X-100 (0.1%) was used as a positive control, and 0.9% (wt/vol) NaCl solution was used as a negative control. Results are expressed as absorbance at 600 nm wavelength. (**B**) Survival rates of mice infected with isolates. (**C**) Dynamic changes in abscess area following inoculation with ST965-MRSA and USA300-LAC in a mouse skin infection model (7-day follow-up observation). ****P < 0.0001.

The driving factors of dominant HA-MRSA clones include high level of virulence, colonization ability, invasive capacity, immune evasion, and other factors. Given that ST965-MRSA represents a potentially globally disseminating clone, we selected four representative ST965-MRSA isolates based on phylogenetic analysis results (covering diversity in *spa* type, resistance phenotypes, and other characteristics) and conducted a comprehensive phenotypic characteristic analysis on them. As shown in Fig. 7A, the hemolytic activity of ST965-MRSA was significantly lower than that of the hypervirulent strain USA300-LAC (*P <* 0.0001) but higher than that of the ST5 strain belonging to the same CC5 clonal complex. To further evaluate the differences in strain virulence *in vivo*, we established the mouse bloodstream infection model and monitored survival curves (Fig. 7B). The results showed that the lethality of ST965-MRSA in mice was intermediate between ST5 and ST8 strains, which was consistent with its hemolytic activity phenotype. Given that *S. aureus* is an important pathogen of skin and soft tissue infections, we further evaluated the pyogenic capacity of each strain through subcutaneous inoculation (Fig. 7C). Compared with USA300-LAC, the abscess area of ST965-MRSA was significantly reduced.

### ST965-MRSA possesses strong invasion and immune evasion capabilities

**Fig 8:**
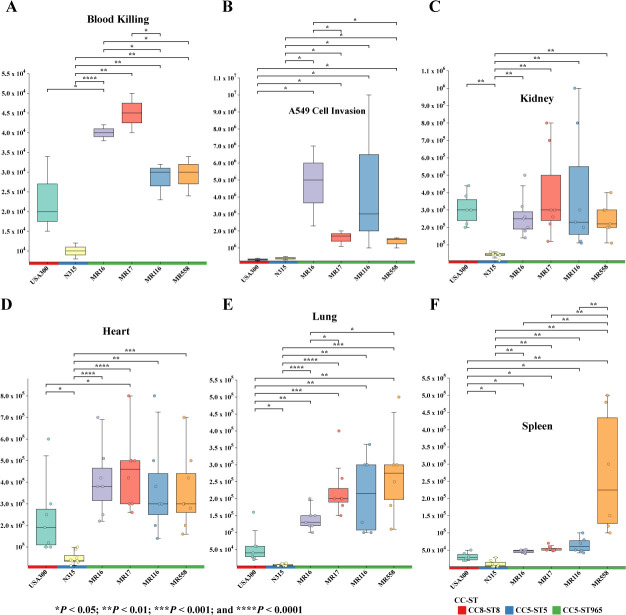
Phenotypic assays of ST965-MRSA. (**A**) Survival assessment of ST965-MRSA in human whole blood bactericidal assay, with results expressed as bacterial CFU. (**B**) Invasion capacity assessment of ST965-MRSA into A549 pulmonary epithelial cells, with results presented as CFU enumeration of internalized bacteria. (**C**) The invasive ability of isolates on mouse kidney. (**D**) The invasive ability of isolates on mouse heart. (**E**) The invasive ability of isolates on mouse lungs. (**F**) The invasive ability of isolates on mouse spleen. *P < 0.05, **P < 0.01, ***P < 0.001, ****P < 0.0001.

CC5 represents a dominant clonal complex in nosocomial infections, exhibiting robust environmental adaptation capabilities (33, 34). As a member of CC5, ST965 may possess similar biological characteristics. The persistent infection ability of bacteria mainly depends on their immune evasion mechanisms and tissue invasion capabilities. Accordingly, we initially utilized the whole blood killing assay to assess the host immune evasion capability of ST965-MRSA. As shown in Fig. 8A, the survival rate of ST965-MRSA in whole blood was significantly higher than that of ST5 and ST8 (*P <* 0.05). To further evaluate the tissue invasion and intracellular survival ability of ST965-MRSA, we conducted *in vitro* cell invasion experiments and *in vivo* infection model studies. In the human lung adenocarcinoma A549 cell invasion model, the intracellular survival rate of ST965-MRSA was significantly higher than that of ST5 and ST8 (Fig. 8B, *P <* 0.05). In the mouse bloodstream infection model, we detected bacterial loads in various organs after infection. Results revealed that ST965-MRSA demonstrated significantly elevated colonization levels in lungs, heart, and spleen tissues compared to ST8 and ST5 (Fig. 8D through F, *P <* 0.05). The above results indicate that the advantages of ST965-MRSA may be more reflected in its *in vivo* survival and tissue invasion ability, representing an infection strategy that favors persistence rather than acute toxicity.

## DISCUSSION

ESKAPE consists of *Enterococcus faecium*, *Staphylococcus aureus*, *Klebsiella pneumoniae*, *Acinetobacter baumannii*, *Pseudomonas aeruginosa*, and *Enterobacter* spp. As an important member of ESKAPE pathogens, *S. aureus* features extensive host range and environmental adaptability, contrasting with the predominantly nosocomial *Acinetobacter baumannii* or the limited zoonotic potential of *Pseudomonas aeruginosa* (35, 36). Like its ESKAPE cousins *Streptococcus*/*Enterococcus* capable of utilizing exogenous fatty acids (37, 38), *S. aureus*, including MRSA, programs a bipartite FakA/B kinase system to scavenge host fatty acids for its full infectivity (39), whereas this is contrary to that of the gram-negative bacterium (esp. *Vibrio species*) developing a unique acyl-ACP synthetase (termed AasS) machinery for activation of exogenous fatty acids (40, 41). Since AasS can be targeted (42, 43), we asked an open question of if MRSA can be leveraged by developing lead compounds against the FakA/B system. Very recently, biotin enzymes are validated as a drug target against certain ESKAPE agents, like *K. pneumoniae* (44, 45) and *P. aeruginosa* (44, 46). Because MRSA encodes an alternative biotin pathway, we anticipated certain biotin enzymes functioning as antivirulence targets against MRSA, while it needs further exploration. Our findings demonstrated that ST965-MRSA challenges the conventional boundaries between CA-MRSA and HA-MRSA by incorporating the SCC*mec* IV element, multiple drug resistance plasmid, and enhanced tissue invasion and immune evasion capacities. The SCC*mec* IV element is widely distributed in CA-MRSA due to its simple structure (typically carrying only the *mecA* gene) and low fitness cost characteristics (47). However, ST965-MRSA, through the acquisition of the pYF965 plasmid, supplemented multiple resistance genes *aac(6′)-Ie*, *blaI-blaR-blaZ*, *erm(B)* and *erm(C)*, significantly enhancing its survival advantage in hospital environments with high antibiotic selection pressure. ST965-MRSA displays considerable genetic plasticity, suggesting its capacity for dissemination across diverse ecological niches (48). The emergence of ST965-MRSA warns us that CA-MRSA can rapidly evolve into a dominant pathogen in hospital environments through the acquisition of key adaptive elements. Consequently, establishing an integrated surveillance system encompassing human, animal, and environmental sectors is essential for effectively monitoring and controlling the dissemination of this emerging lineage.

ST965-MRSA is deficient in *pvl* and *tsst-1* genes, with the *hlb* gene truncated by phage integration. However, the loss of key virulence factors does not necessarily equate to reduced pathogenicity. Instead, pathogens may maintain or even enhance their infectious capacity through alternative strategies. For instance, *Streptococcus suis* utilizes the fak system to hijack host-derived fatty acids to enhance its persistent infection capability (37, 38). Further phenotypic results showed that ST965-MRSA acquired enhanced persistence capabilities at the expense of acute virulence. This approach minimizes host inflammatory reactions, thus circumventing the initiation of intensive clinical interventions while preserving the capacity for opportunistic infections, constituting an optimal dissemination strategy within healthcare settings. This trade-off between virulence and persistence may explain the increasing phenomenon of refractory MRSA persistent infections in current hospital environments while also revealing the limitations of traditional virulence indicators in predicting clinical outcomes (49). Notably, the phenotypic characteristics of ST965-MRSA showing reduced acute virulence and enhanced persistent infection ability may represent a common evolutionary strategy of hospital-acquired pathogens, and medical institutions should continuously track the transmission dynamics and evolutionary trajectories of such adaptive clones.

This study confirms that ST965-MRSA carries a high proportion of IEC, making these factors candidates for diagnostic markers and therapeutic targets (49). The staphylococcal chemotaxis inhibitory protein (CHIPS, encoded by *chp*), as an important extracellular protein, can effectively inhibit the chemotaxis of neutrophils and monocytes, making it a primary candidate target for developing neutralizing antibodies (50). By blocking the function of CHIPS, such therapies can restore the host innate immune response and improve bacterial clearance in bloodstream infections. Similarly critical for pathogenesis, staphylokinase (SAK, encoded by *sak*) is a plasminogen activator secreted by the majority of *S. aureus* isolates (51). SAK promotes bacterial tissue invasion and dissemination at sites including the skin by activating plasminogen and producing plasmin (52). Meanwhile, some studies indicate that SAK can inhibit biofilm formation, making SAK a double-edged sword that must be handled carefully to avoid inadvertently promoting biofilm-associated infections. Staphylococcal complement inhibitor (SCIN, encoded by *scn*) can interact with C3 convertase, and by blocking SCIN, C3b deposition increases, restoring the opsonophagocytic function of the complement system (53). As medical advances have significantly prolonged the survival of immunocompromised patients, developing specific vaccines and monoclonal antibodies targeting immune evasion factors has become an urgent clinical need.

A separate, yet particularly intriguing observation from this study is the association between a RiPP-like BGC and gentamicin resistance. It is well known that aminoglycoside entry into cells depends on cell membrane potential (54), and RiPPs possess membrane activity that can alter membrane permeability or interfere with ion gradients. Based on these established mechanisms, we hypothesize that RIPP-like BGCs produced by ST965 may interact with cellular membranes, potentially resulting in membrane potential disruption and thereby diminishing gentamicin uptake (55). To validate this hypothesis and elucidate the underlying mechanisms, additional studies are required to focus on the effects of this specific peptide on membrane potential and gentamicin uptake.

Although our study provides important insights into the biological characteristics of ST965-MRSA, certain limitations still exist. The relatively limited sample size and the geographic concentration of isolates from China may not fully capture the global genomic diversity and epidemiological landscape of ST965-MRSA lineage. To address this limitation, we advocate for establishing an international consortium and global surveillance network for ST965-MRSA, which would enhance genomic and epidemiological data sharing, enabling deeper insights into its evolution, transmission, and pathogenesis.

In conclusion, the emergence of ST965-MRSA exemplifies a novel paradigm in MRSA adaptive evolution. This clone has successfully accomplished ecological niche adaptation within nosocomial environments through the modulation of virulence, antimicrobial resistance, and survival capacities. This evolutionary approach challenges conventional perspectives on MRSA epidemiology, underscoring the imperative for sustained genomic monitoring, novel therapeutic interventions, and adaptive infection control measures. In the context of increasingly severe antimicrobial resistance, comprehensive understanding of the pathogenic features of emerging clones like ST965-MRSA is essential for effectively preventing and treating healthcare-associated infections.

## SUPPLEMENTAL MATERIAL

Supplemental material msystems.00798-25-s0001.docx Fig. S1 and Table S1.

ASM does not own the copyrights to Supplemental Material that may be linked to, or accessed through, an article. The authors have granted ASM a non-exclusive, world-wide license to publish the Supplemental Material files. Please contact the corresponding author directly for reuse.
