# Supplementary material for: Genomic and phenotypic characterization of methicillin-resistant Staphylococcus aureus ST965: an emerging hospital-adapted clone with enhanced invasiveness
Source: mSystems. 2025 Jul 31;10(8):e00798-25. doi: 10.1128/msystems.00798-25 (PMC12364385; doi:10.1128/msystems.00798-25)
Supplement: Supplemental material — Fig. S1 and Table S1. [file msystems.00798-25-s0001.docx]

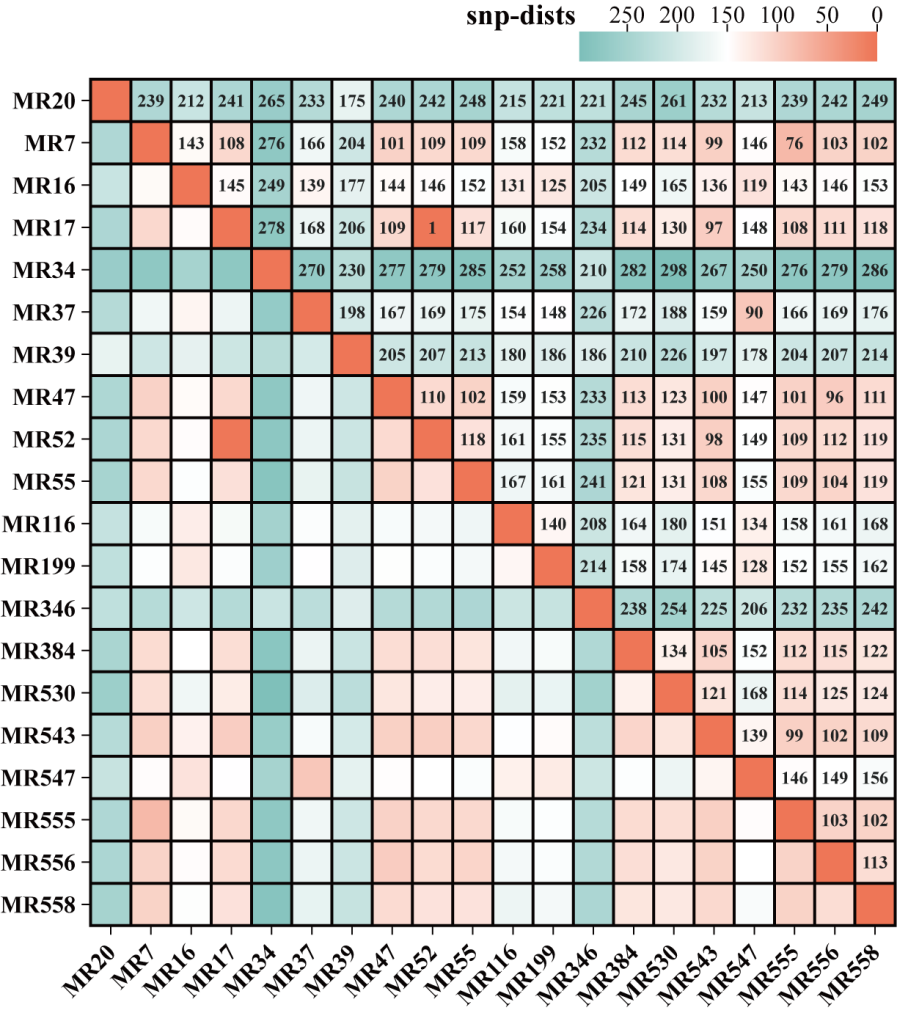


**Fig S1.** The number of single nucleotide polymorphisms (SNPs) between each isolate.

| Table 1 The genomic characteristics of the STs of CC5 | | | | | | | | | |
| --- | --- | --- | --- | --- | --- | --- | --- | --- | --- |
| **CC** | **ST** | **SCCmec type** | **Predominant spa type** | **Predominant IEC type** | **IEC genes** | **Predominant toxin factors profiles** | **Predominant Adhesins factors** | **agr type** | **PVL genes** |
| CC5 | ST5-MRSA | II | t2460 | none | *-* | sec-sell-tsst1 | clfB，fnbA | II | *-* |
|  | ST105-MRSA | II | t002 | B | *sak-chp-scn* | none | clfB，fnbA，fnbB | II | - |
|  | ST764-MRSA | II | t1084, t002 | B | *sak-chp-scn* | seb | clfA，clfB，fnbA | II | *-* |
|  | ST965-MRSA | IV | t062 | A | *sea-sak-chp-scn* | *sea* | clfA，clfB，fnbA | II | *-* |

**Table 1**. The genomic characteristics of the STs of CC5
